# Inflammation and emphysema in cigarette smoke-exposed mice when instilled with poly (I:C) or infected with influenza A or respiratory syncytial viruses

**DOI:** 10.1186/s12931-016-0392-x

**Published:** 2016-07-01

**Authors:** Yohannes A. Mebratu, Kevin R. Smith, Getahun E. Agga, Yohannes Tesfaigzi

**Affiliations:** COPD Program, Lovelace Respiratory Research Institute, 2425 Ridgecrest Drive SE, Albuquerque, NM 87108 USA; Agricultural Research Service, U.S. Department of Agriculture, R, Clay Center, Nebraska, USA

**Keywords:** Influenza A virus, Respiratory syncytial virus, Poly (I:C), Emphysema, Mice, Cigarette smoke, Mouse

## Abstract

**Background:**

The length of time for cigarette smoke (CS) exposure to cause emphysema in mice is drastically reduced when CS exposure is combined with viral infection. However, the extent of inflammatory responses and lung pathologies of mice exposed to CS and infected with influenza A virus (IAV), respiratory syncytial virus (RSV), or treated with the viral derivative dsRNA (polyinosine-polycytidylic acid [poly (I:C)] have not been compared.

**Methods:**

Mice were exposed to CS or filtered air for 4 weeks and received a single dose of vehicle, AV, or RSV infection and extent of inflammation and emphysema was evaluated 14 d later. In another set of experiments, mice were instilled with poly (I:C) twice a week during the third and fourth weeks of CS exposure and immediately analyzed for extent of inflammation and lung pathologies.

**Results:**

In CS-exposed mice, inflammation was characterized mainly by macrophages, lymphocytes, and neutrophils after IAV infection, mainly by lymphocytes, and neutrophils after RSV infection, and mainly by lymphocytes and neutrophils after poly (I:C) instillations. Despite increased inflammation, extent of emphysema by poly (I:C) was very mild; but was robust and similar for both IAV and RSV infections with enhanced MMP-12 mRNA expression and TUNEL positivity. Both IAV and RSV infections increased the levels of IL-17, IL-1β, IL-12b, IL-18, IL-23a, Ccl-2, Ccl-7 mRNAs in the lungs of CS-exposed mice with IAV causing more increases than RSV.

**Conclusion:**

CS-induced inflammatory responses and extent of emphysematous changes differ depending on the type of viral infection. These animal models may be useful to study the mechanisms by which different viruses exacerbate CS-induced inflammation and emphysema.

## Background

Chronic obstructive pulmonary disease (COPD), including emphysema and chronic bronchitis, is the third leading cause of death in the US [1]. COPD refers to a broad group of lung diseases with airflow limitation, parenchymal destruction, fibrosis around small airways involving several different cells (neutrophils, macrophage, CD8 lymphocytes) [2, 3], and small-airway obstruction [4]. The most studied of these conditions is emphysema, which is characterized by permanent inflammation and irreversible destruction of alveolar walls, leading to airspace enlargement and loss of elastic recoil and hyperinflation [5]. Exposure to CS accounts for about 80 % of all COPD cases [6–9], but other factors when combined with cigarette smoke play a role in exacerbating CS-induced COPD [10, 11]. Repeated incidences of COPD exacerbations are associated with more rapid disease progression, poorer quality of life, and higher risk of mortality [12, 13]. Environmental pollutants, including wood smoke [10, 14, 15–17] and diesel exhaust [18] play a role in enhancing CS-induced inflammation. Bacterial and viral infections are also major causes for COPD exacerbations [19–25] leading to decline in lung function and concomitant acute deterioration in respiratory health [12]. Interestingly, as many as 40 to 60 % of all exacerbations are attributed to respiratory viral infections alone [26, 27].

Interactions between CS exposure and viral infection induce exaggerated inflammatory and tissue remodeling responses [28, 29]. Viral infections, such as IAV [30–32] and respiratory syncytial virus (RSV) [19, 24, 25] are associated with acute COPD exacerbations, but the severity of infection in patients with COPD depends on both viral and host factors [33]. For example, RSV accounts for a quarter of all viral-induced COPD exacerbations [19, 24, 25] and is an important respiratory pathogen in the elderly, particularly in those with chronic lung diseases such as COPD [23–25]. Approximately 60 million people currently smoke cigarettes in the US alone, and many of the immunocompromised elderly individuals are susceptible to RSV infections and will have recurrent seasonal flu infections. To understand disease progression and develop better therapies, establishing animal models that reflect these conditions in humans is crucial.

While the development of emphysema in mice requires an exposure to CS for 4–8 months [34–38], infection with respiratory viruses enhances CS-induced inflammation and causes emphysema within 6 weeks [28].

The purpose of the current study was to establish mouse models that recapitulate the cardinal features of COPD within a short period of time by comparing extent of inflammation and emphysema after a 4 week CS exposure in combination with a single infection of either IAV, RSV, or multiple instillation with Poly(I:C). We show that such a 2-hit system enhances lung inflammation and the development of emphysema. Despite these similarities, these animal models also showed major differences in the type and extent of inflammation and alveolar destruction.

## Methods

### Cigarette smoke exposure and virus infection

**Fig. 1:**
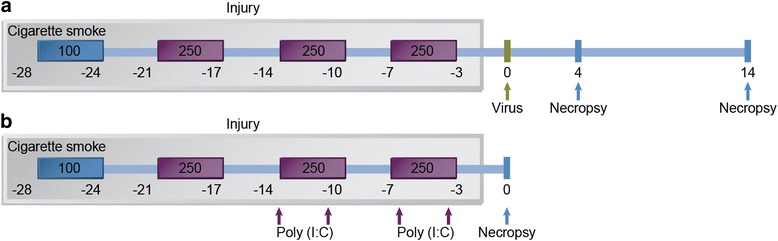
Schematic diagram of the study design for cigarette smoke exposure and infection with IAV or RSV or Poly (I:C) treatment. C57BL/6 mice were first exposed to mainstream cigarette smoke (6 h/day, 5 days/week; 1 week at 100TPM/m^3^ and 3 weeks at 250TPM/m^3^) (CS) or filtered air (FA). **a** Mice were intranasally instilled with vehicle or 5 × 10^3^ pfu IAV (IAV) or 4 × 10^6^ pfu RSV (RSV) and lung tissues were harvested at 4 or 14 days post infection. **b** Mice were instilled twice a week with 50 μl of poly (I:C) (50 μg/50 μl) on the third and fourth week of CS exposure and lung tissues were harvested at the end of the 4 weeks of exposure

Previous studies have shown that female mice are more susceptible to CS-induced lung injury and develop emphysema earlier than male mice from the same strain [37]. Therefore, 6 to 8 week old female C57BL/6 mice were purchased from Jackson Laboratories (Bar Harbor, ME) and housed in a pathogen-free facility. Mice were exposed in whole body Hazelton 1000 chambers as described [37] using Type 2R4F research cigarettes (Kentucky Tobacco Research and Development Center). A computer-based system provided 24-h monitoring and recording of airflow rate, temperature, humidity, and pressure of the exposure chambers. Previous studies have shown that infection with up to 10^5^ pfu of mouse-adapted strain of H3N3-HKx31 IAV or 10^7^ pfu of RSV [39] cause significant inflammatory responses in the lungs of C57BL/6 mice without causing fatality for at least 14 days [40, 41] (and personal observation). Because infection was going to be combined with exposure to CS, we used 5 × 10^3^ pfu H3N3-HKx31 IAV or 4 × 10^6^ pfu RSV to account death of mice that may already have lost weight after 4 weeks of CS exposure. To reduce mortality, mice were first conditioned to CS by exposing them to 100 mg total particulate matter (TPM)/m^3^ (6 h/day, 5 days/week) for the first week and then to 250 mg TPM/m^3^ (6 h/day, 5 days/week) over weeks 2–4. Mice were then intranasally instilled with VEHICLE (FA-Vehicle or CS-Vehicle) or 5 × 10^3^ pfu of mouse-adapted strain of H3N3-HKx31 IAV (FA-IAV or CS-IAV) or 4 × 10^6^ pfu RSV (FA-RSV or CS-RSV), and euthanized at day 14 post viral challenge (Fig. 1a). In the Poly(I:C) study group, mice were exposed to FA or CS for 6 h/day, 5 days/week, and during the third and fourth week of CS exposure instilled twice a week with 50 μl of VEHICLE (FA-Vehicle or CS-Vehicle) or poly(I:C) (FA-Poly(I:C) or CS-Poly(I:C)) (50 μg/50 μl) as described [28], and sacrificed at the end of the 4 weeks of CS exposure (Fig. 1b). All studies were conducted at Lovelace Respiratory Research Institute, a facility approved by the Association for the Assessment and Accreditation for Laboratory Animal Care International. All experiments were pre-approved by the Lovelace Respiratory Research Institute Institutional Animal Care and Use Committee (IACUC), and the Environmental Safety and Health department (ES&H) with protocol reference numbers FY11-006 and FY09-024.

### Lung inflammatory cells, histology and morphometry

Mice were euthanized by lethal injection of pentobarbital sodium followed by exsanguination. Lung inflammatory cells were recovered by bronchoalveolar lavage (BAL) and total recovered cells were counted in BAL fluid (BALF) and immune cell types in the BALF were quantified after staining cytospins using a Diff-quik kit (Siemens Healthcare Diagnostics, Inc., Newark, DE) per manufacturer’s direction. For lung histology and morphometry, lungs were inflated and fixed with 4 % buffered paraformaldehyde at a constant hydrostatic pressure of 25 cm for 4 h and further immersed in fixative for 48 h. The inflated lungs were embedded in paraffin and 5 μm sagittal sections were stained with hematoxylin and eosin (H&E) for histological evaluation as described [42]. To analyze inflammation-associated lung damages, H&E–stained lung tissues were evaluated in a blinded manner with a semi-quantitative system according to the relative degree of inflammation and tissue damage. Inflammatory changes were evaluated and described by a board certified pathologist. Lung inflammatory changes were graded based on the following parameters: peri-bronchiolar and bronchial infiltrates, bronchiolar and bronchial luminal exudates, perivascular infiltrates, parenchymal pneumonia, and edema, as previously described [43–46]. Each parameter was graded on a scale of 0–4 with 0, absent; 1, slight; 2, mild; 3, moderate; and 4, severe. The cumulative scores of inflammatory infiltration, degeneration and necrosis provided the total score per animal, and the average score of mice in each group was taken as total score for that group. Morphometric analysis of alveolar volume measurement was carried out using the Visiomorph module of VisioPharm analysis software (Visiopharm, Hoersholm, Denmark).

### TUNEL assay

We analyzed fixed lung tissues from each group for cells with internucleosomal DNA fragmentation using the TUNEL assay, as described elsewhere [47, 48]. Briefly, terminal deoxynucleotidyltransferase was used to incorporate biotin-16-dUTP into the ends of DNA fragments. For lung tissues from each group with internucleosomal DNA fragmentation TUNEL signals were visualized using the Vectastain avidin-biotin complex kit and the peroxidase substrate diaminobenzidine (Vector Laboratories, Burlingame, CA) and microscopy (Zeiss) as described by the manufacturer.

### Analysis of mRNA levels

Total RNA was isolated from snap-frozen lung tissue using the RNeasy mini kit (Qiagen, Valencia, CA) which included a 10 min on-column DNAse treatment. Mouse IL17 RT^2^ Profiler PCR array (SA Biosciences, Valencia, CA) was used to analyze host gene expression on samples from lungs of mice exposed to FA or CS and infected with IAV or RSV. SA Biosciences web based RT^2^ profiler PCR array data analysis program was used to calculate fold changes. Quantitative TaqMan RT-PCR was performed following manufacturer’s instructions (Applied Biosystems, Foster City, CA) to measure mRNA levels for IL-17a, IL-17c, IL-17d, IL-17 f. IL-1β, IL-12b, IL-18, IL-23a, Ccl-2, Ccl-7 at day 4 post infection in lung tissue samples from Vehicle- or IAV- or RSV-infected mice exposed to FA or CS. Quantitative RT-PCR analysis for MMP-12 mRNA was performed at day 14 post infection. Viral load was evaluated by quantitative RT-PCR using TaqMan primer and probe that detects IAV M and RSV NS1 gene mRNAs. RT-PCR was performed in an ABI Prism 7900 Sequence Detection System (Applied Biosystems) using universal thermal cycling parameters. Gene expression was analyzed using the comparative C_T_ method after normalization to GAPDH of Vehicle infected, C-exposed mice.

### Data analysis

Data were analyzed and graphed using GraphPad PRISM version 5.04. Data were generally analyzed using one-way ANOVA, and when significance was detected, the Fisher’s exact least significant difference test was used to determine differences between groups. Body weight comparisons were analyzed using ANOVA with repeated measures, followed by a post hoc Fisher’s least significance difference test. Viral titres in the lung were compared using an unpaired Student’s two-tailed *t*-test. Comparisons of inflammatory cells, weighted mean alveolar volume, mean linear intercepts, inflammation scores, MMP-12 RNA levels and TUNEL positivity among groups were analyzed using ANOVA followed by Tukey’s Multiple Comparison Test. Unless otherwise stated data are presented as mean +/− standard error of mean (SEM) for at least 6 mice/group. Probability values less than 0.05 were considered significant.

## Results

### Lung inflammation in CS-exposed mice when infected with IAV and RSV

**Fig. 2:**
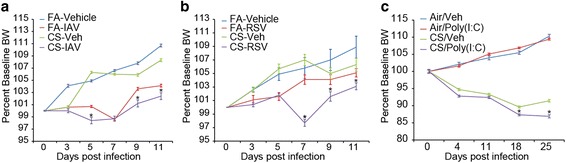
C57BL/6 mice exposed to FA or CS for 4 weeks, intranasally instilled with vehicle or (**a**) IAV or (**b**) RSV and euthanized at day 14 post viral challenge. Percent changes in body weights were compared at different time points. **c** C57BL/6 mice were exposed to FA or CS for 4 weeks, instilled twice a week with 50 μl of poly(I:C) (50 μg/50 μl) or vehicle on the third and fourth week of FA (poly(I:C)FA) or CS (poly(I:C)CS) exposure. Percent changes in body weight were compared. *N* = 6 mice per group * *p* < 0.05

**Fig. 3:**
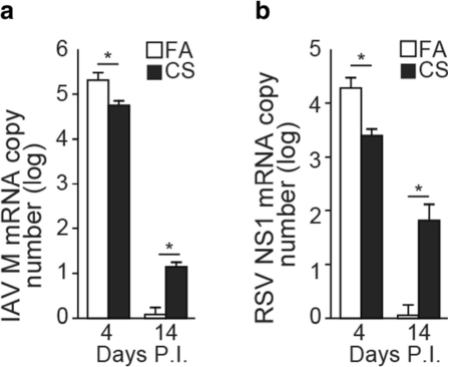
IAV and RSV infections persist longer in CS-injured lungs. Viral load was compared in the lung tissues of FA- or CS-exposed mice on d 4 and 14 of infection with (**a**) IAV or (**b**) RSV using qRT-PCR. Total RNA was isolated from snap-frozen lung tissues and quantitative TaqMan RT-PCR analysis was performed to assess IAV M1 or RSV NS1 gene expression. Samples were normalized to mouse GAPDH RNA levels. *N* = 6 mice per group; data reported as mean ± SEM. *p < 0.05

To study the relative significance of different respiratory viruses we first compared changes in body weight over time after infection. Previous studies reported that CS exposure reduces the body weight of mice [11, 37]. We found that IAV significantly reduced body weight in both FA- and CS-exposed mice (Fig. 2a). However, RSV infection or instillation with the viral derivative Poly (I:C) affect the body weight in CS-exposed but not in FA-exposed mice (Fig. 2b and c). We compared the viral load in the lung tissues of FA-IAV/RSV or CS-IAV/RSV mice on d 4 and 14 of infection using qRT-PCR and found that both IAV M and RSV NS1 gene copy numbers were significantly reduced in CS-IAV/RSV compared to FA-IAV/RSV lung tissues at 4 d post infection. However, the viral load was much higher in CS-IAV/RSV groups compared to FA-IAV/RSV mice in both IAV- and RSV-infected groups at 14 d post infection although the overall viral load was lower in both FA and CS-exposed mice compared to that found at 4 d (Fig. 3a and b).

**Fig. 4:**
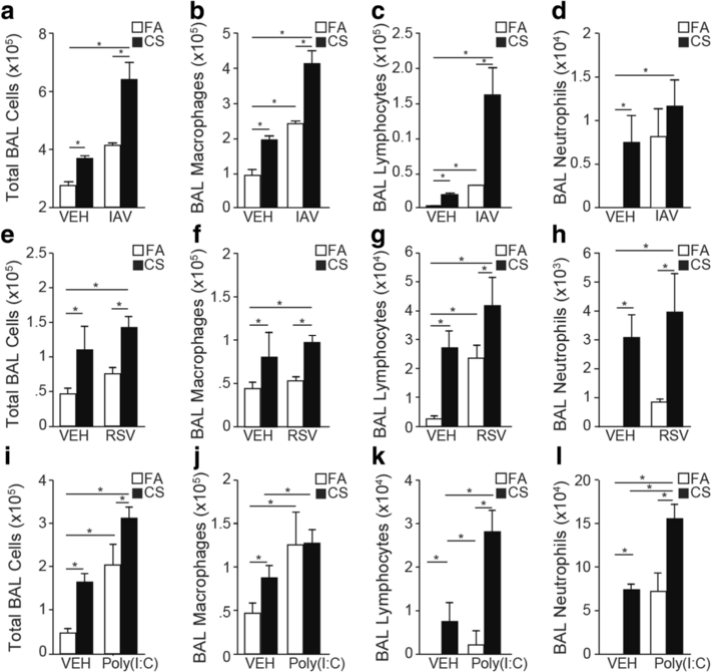
CS injury alters inflammatory cell recruitment in response to IAV, RSV, or Poly (I:C). BAL was performed on (**a**-**h**) FA-Vehicle, CS-Vehicle, FA-IAV/RSV and CS-IAV/RSV mice 14 post viral challenge or (**i**-**l**) FA-Vehicle, CS-Vehicle, FA-poly(I:C) and CS-poly(I:C) mice instilled with 50 μl of poly (I:C) (50 μg/50 μl) or vehicle twice a week on the third and fourth week of CS exposure. Total cell counts (**a**, **e**, **i**), and differential analysis for macrophages (**b**, **f**, **j**), lymphocytes (**c**, **g**, **k**) and neutrophils (**d**, **h**, **l**) were performed on isolated BAL cells. *N* = 6 mice per group; data reported as mean ± SE; * *p* < 0.05, ** *p* < 0.01, ***p < 0.001

The total number of inflammatory cells recovered in the broncho-alveolar lavage (BAL) increased significantly in CS-Vehicle compared to FA-Vehicle controls (Fig. 4a, e, and i). Exposure to CS caused approximately 2-fold increases in BAL macrophages, which were further increased by IAV and poly(I:C) but not RSV (Fig. 4b, f, and j). Overall, both RSV or poly(I:C) showed significantly lower macrophage numbers in the BAL (Fig. 4b, f, j). FA-IAV or FA-RSV mice had significantly increased number of BAL lymphocytes compared to FA-Vehicle mice, but the increases in BAL lymphocytes by poly(I:C) were not significant. However, CS exposure significantly enhanced the number of BAL lymphocytes in IAV, RSV- or poly (I:C)-treated groups (Fig. 4c, g, and k). Exposure to CS increased the number of BAL neutrophils in vehicle-treated mice. The BAL neutrophil levels were increased by infection with IAV, RSV or poly(I:C) alone, which were further enhanced by exposure to CS (Fig. 4d, h, and l). Relative to their respective CS-Vehicle groups, CS-poly(I:C) compared to CS-RSV and CS-IAV caused the most neutrophilic inflammation (Fig. 4l). Together, these results suggest that, in CS-injured lungs, while IAV infection had a synergistic effect in increasing the number of macrophages and lymphocytes and slightly neutrophils, RSV mainly caused further increases in lymphocytes and somewhat for neutrophils, and poly(I:C) primarily caused increases in lymphocytes and neutrophils.

**Fig. 5:**
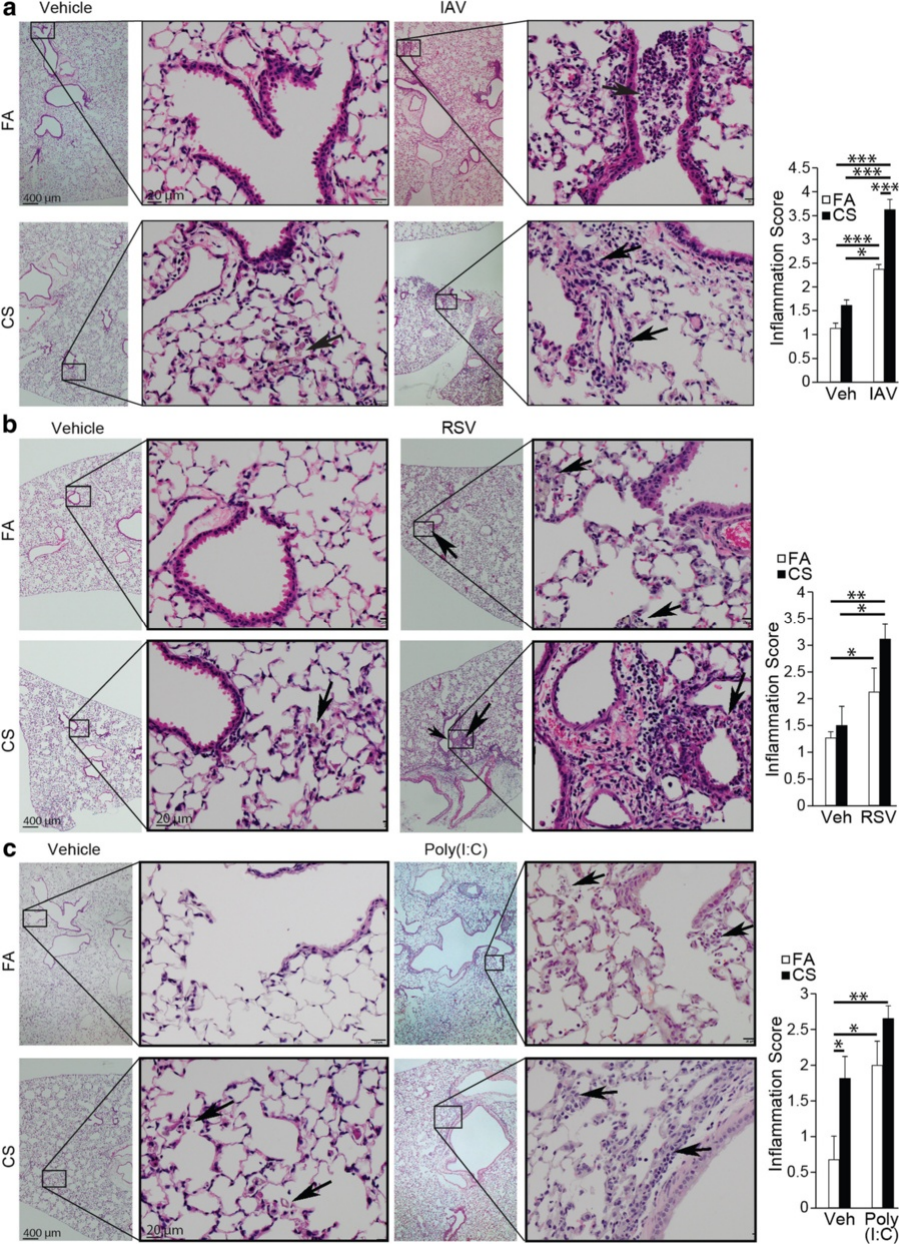
Inflammation score in H&E-stained lung tissues from (**a**) IAV-infected or (**b**) RSV-infected or (**c**) Poly (I:C) instilled mice with or without exposure to CS on day 14 post infection. The arrows in FA-IAV image indicate neutrophilic and monocytic infiltrates at the bronchovascular junction, in the CS-Vehicle image indicate alveolar macrophage accumulation, in CS-IAV indicate infiltrates of monocytes, lymphocytes and neutrophils. The arrows in FA-RSV indicate infiltrates of lymphocytes and some macrophages, in CS-RSV images indicate infiltrates of lymphocytes and monocytes around the vessels, and neutropils and necrotic cell debris in the airways. The arrows in FA-poly(I;C) and CS-Poly(I:C) images indicate infiltrates of macrophages, and lymphocytes with some necrosis. Inflammation scores were evaluated as described in MATERIALS AND METHODS. *N* = 6 mice per group; data reported as mean ± SE; * *p* < 0.05, ** *p* < 0.01, ***p < 0.001

Lung inflammatory changes were graded in H&E stained lung tissues. Generally, smoke alone caused mild inflammation consisting of mild peri-bronchovascular, broncho-alveolar and septal alveolar macrophage accumulation with some pigmentation. FA-IAV (Fig. 5a) or FA-RSV (Fig. 5b) caused enhanced inflammation with prominent inflammatory cells surrounding the alveoli, bronchi and the parenchyma with FA-IAV showing more of neutrophilic and monocytic infiltrates at the broncho-vascular junction and FA-RSV showing mainly infiltrates of lymphocytes with some macrophages. The lung tissues from FA-poly(I:C) and CS-Poly(I:C) showed infiltrates of macrophages, neutrophils, lymphocytes and some necrosis, with more severe infiltration of inflammatory cells in the CS-poly(I:C) group (Fig. 5c). Compared to FA-IAV or FA-RSV mice, inflammation was more pronounced in the lung tissues of CS-IAV (Fig. 5a) and CS-RSV mice (Fig. 5b), respectively. When compared to FA-poly(I:C) group, inflammation was also significantly increased in the lung tissues of CS- poly(I:C) mice; however, inflammation was milder compared to CS-IAV- or CS-RSV groups (Fig. 5c). Alcian blue/periodic acid Schiff (AB/PAS) staining showed that there were some mucous cells in the airways of FA-IAV, FA-RSV, or FA-poly(I:C) treated mice with some increases in CS-IAV, CS-RSV, and CS-poly(I:C) groups. However, no major differences in mucous hypersecretion between the three different groups was observed (data not shown).

### Infection with IAV or RSV leads to the development of emphysema in CS-exposed lung

**Fig. 6:**
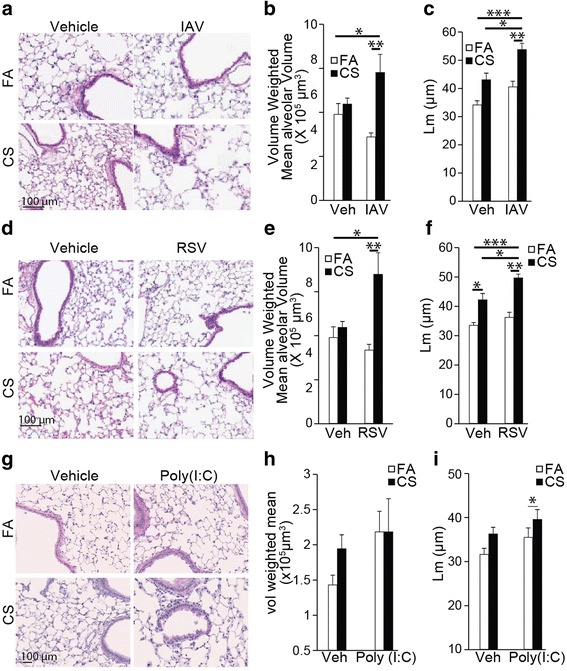
Alterations in alveolar structure and volume by IAV, RSV or Poly (I:C) in the CS-exposed lungs. **a**-**f** Mice were exposed to FA or CS for 4 weeks and instilled with vehicle or (**a**-**c**) IAV or (**d**-**f**) RSV, and euthanized at day 14 post viral challenge. **g**-**i** C57BL/6 mice were exposed to FA or CS for 4 weeks, instilled twice a week with 50 μl of poly(I:C) (50 μg/50 μl) or vehicle on the third and fourth week of CS exposure. Mice were euthanized at the end of 4 weeks of FA or CS exposure. Representative images (**a**, **d**, **g**), morphometric analysis for volume weighted mean alveolar volume (**b**, **e**, **h**), and mean alveolar chord length (**c**, **f**, **i**) of lungs inflated and fixed with formalin under constant pressure. *N* = 8 mice per group; data reported as mean ± SE; *p < 0.05, **p < 0.01, ***p < 0.001

Morphometric analysis on H&E-stained lung sections showed that IAV (Fig. 6a, b, and c) or RSV (Fig. 6d, e and f) caused significant increases in volume weighted mean alveolar volume (Fig. 6b and e) and mean linear intercepts (Lm) (Fig. 6c and f) in mice exposed to CS at day 14 post infections. While poly(I:C) showed no significant increase in the weighted mean alveolar volume, the mean linear intercept (Lm) was significant increase (Fig. 6g, h, i), suggesting that the difference to vehicle and CS-exposed groups was minimal.

### IAV and RSV infections increase the expression of IL-17 and its upstream regulators

**Fig. 7:**
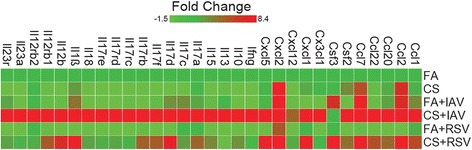
IAV or RSV infections induce IL-17 in cigarette smoke-injured lung. C57BL/6 mice were exposed for 4 weeks to FA or CS and infected with Vehicle or IAV or RSV. RNA was isolated from lung tissues and IL17 RT^2^ Profile PCR array was performed for IL-17 regulated genes at day 4 p.i. All samples were normalized to mouse GAPDH RNA levels. Heat map blocks represent average RNA levels *N* = 6 mice per group and are normalized to FA samples

Because the poly(I:C) group showed minimal change in emphysema, we continued further analyses with the IAV and RSV-infected mice. Previous studies have shown that IL-17, an important mediator of CS-induced emphysema, induces the expression of inflammatory cytokines and matrix degrading proteinases MMP-9 and MMP-12 [49]. We found that infection with IAV or RSV caused an altered inflammatory response in FA-exposed or CS-injured lungs. IAV and RSV infection increased the expression of more than 26 and 16 cytokine-encoding genes in the lungs of CS-exposed mice compared to FA-exposed mice, respectively (Fig. 7). Therefore, we selected specific IL-17-related cytokines and chemokines and further investigated whether IAV or RSV have synergistic effects to enhance the expression these genes in CS-exposed lungs. IAV or RSV infection enhanced lung mRNA levels for IL-17a, IL-17c, IL-17d, IL-17f, IL-1β, IL-12b, IL-18, IL-23a, Ccl-2, and Ccl-7 in CS-exposed mice. However, IAV caused more increases in the IL-17-related cytokines and chemokines tested than RSV in CS-exposed mice (Table 1).

**Table 1:** IAV and RSV alter cigarette smoke induced lung cytokine mRNA expression

Cytokine^a^	IAV	RSV
IL-17a	994.017	4.5291
IL-17c	24.771	3.912
IL-17d	57.981	9.4289
IL-17f	221.587	9.5407
IL-1β	14.3941	4.59729
IL-12b	58.7896	9.9
IL-18	94.4269	3.8055
IL-23a	294.226	3.0546
Ccl-2	37.183	4.95625
Ccl-7	90.9844	22.2711

^a^mRNA isolated at day 4 post infection was analyzed by qRT-PCR and normalized to mouse GAPDH RNA. Data are presented as fold change in CS-IAV/RSV compared with CS-Vehicle groups

### IAV and RSV infections enhance the expression of MMP-12 in cigarette smoke-injured lung

**Fig. 8:**
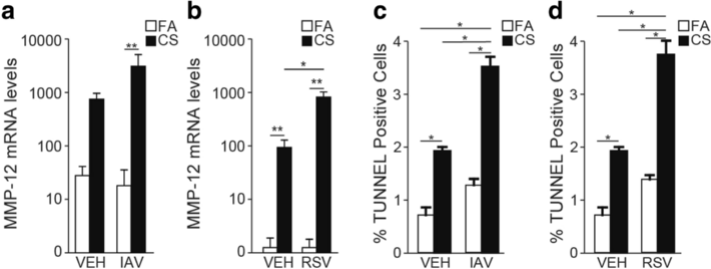
CS injury induces MMP12 in the lungs in response to IAV or RSV. **a**-**b** C57BL/6 mice were exposed for 4 weeks to FA or CS and instilled intranasal with Vehicle, (**a**) IAV or (**b**) RSV. Quantitative RT-PCR analysis for MMP-12 mRNA was performed at day 14 post viral infection. All samples were normalized to mouse GAPDH RNA levels. **c**-**d** Lung tissues from FA- or CS-exposed mice instilled intranasal with vehicle or (**c**) IAV or (**d**) RSV were analyzed for internucleosomal DNA fragmentation using TUNEL assay. *N* = 6 mice per group; data reported as mean ± SE; *p < 0.05; **p < 0.01, ***p < 0.001

The proteinase MMP-12 is an important enzyme in the development and progression and emphysema [50, 51]. Although IAV was used at a lower titer, IAV (Fig. 8a), but not RSV (Fig. 8b) infection increased MMP-12 mRNA levels in FA-exposed mice. Overall, four weeks of exposure to CS compared with FA caused significant increases in the MMP-12 mRNA levels. However, in CS-exposed mice, both IAV (Fig. 8a) and RSV (Fig. 8a) infections synergistically enhanced MMP-12 mRNA expression with IAV infection more than RSV.

Apoptosis plays an important role in the development of emphysema [52–54]. Analyses for internucleosomal DNA fragmentation using the TUNEL assay showed that neither IAV nor RSV infection alone caused significant increase in TUNEL positivity in FA-exposed group. Compared to FA-exposed mice, CS-exposed mice had significant increase in TUNEL positivity in the lung tissues and these increases were further enhanced equally by both IAV (Fig. 8c) and RSV infection (Fig. 8d).

## Discussion

The present study utilized mouse models with existing CS exposure to compare extent of inflammatory responses and tissue destruction when infected with IAV, RSV, or instilled with Poly (I:C). When comparing these three models, one needs to consider the differences in the viral titers for IAV and RSV used to infect the mice. In addition, multiple doses of Poly (I:C) were delivered to mice over the last two weeks of CS exposure, and mice were euthanized right after 4 weeks of exposure to CS and were not kept for an additional 14 days in FA as in the cases with IAV and RSV groups. Repeated administration was chosen to compensate for the lack of replication in the poly(I:C) group.

We found that by day 4 post infection CS-exposed groups had significantly lower copy number of viruses than the FA groups for both IAV and RSV study groups. This may be due to early induction of type I IFN by cigarette smoke [28, 55], which may suppress virus replication. As inflammation progresses, IFN response may wane resulting in delayed viral clearance and more titer in CS-injured lungs than FA-exposed lungs on day 14 post infection.

While the overall total numbers of inflammatory cells recovered in the BAL of CS-IAV/RSV and CS-poly(I:C) mice were synergistically increased, differences in the type and degree of inflammatory responses were observed among these groups. The CS-IAV group displayed increased number of macrophages and lymphocytes with few neutrophils, and CS-RSV group showed increased lymphocytes and neutrophils, similar to that observed in the CS-poly(I:C) group. These findings are consistent with previous studies showing that IAV or poly (I:C) lead to additive effects in the level of BAL inflammatory cells in mice exposed to CS [28, 56]. Interestingly, despite higher titers used for RSV, IAV caused more inflammation suggesting that different viruses have distinct inflammatory patterns either alone or when combined with CS exposure. Macrophages and neutrophils are the main sources of proteases in lungs, and there are correlations between the degree of macrophage and neutrophil inflammation and severity of airflow obstruction [57]. Although significant increases in the BAL neutrophils were observed among the models tested, poly (I:C) caused the mildest form of emphysema in CS-exposed mice. For the poly(I:C) group, emphysema measures were assessed at 4 weeks of CS exposure while the IAV and IAV groups of mice were assessed 14 d later. Further, IAV or RSV alone did not cause increases in alveolar spaces compared to their respective vehicle controls. Hence, it is possible that the poly(I:C) group could develop more pronounced structural changes in CS-exposed mice similar to the virally challenged groups given the appropriate time for the changes to develop. Emphysema that develops in mice after 6 months of exposure remains pronounced even 6 months later [37], and future studies will need to examine the persistence of the observed emphysematous changes several months after the initial infections.

Both IAV and RSV infection induced IL-17a, IL-17b, IL-17d, and IL-17f, IL-1β, IL-12b, IL-18, IL-23, Ccl-2, and Ccl-7 mRNA levels in the lungs of CS-injured mice. CD8^+^ T-lymphocytes are critical for the induction of inflammation and tissue destruction in a murine model of smoke-induced emphysema [58]. CD4^+^ T cells, upon activation and expansion, develop into different T helper cell subsets with different cytokine profiles and distinct effector functions [59]. Thus, IAV or RSV in CS-exposed mice may cause the differentiation of Th17 cells or may affect γδ T cells, NK cells, and neutrophils to produce IL-17 in response to CS exposure dependent induction of IL-1β, IL-18, or IL-23 [60]. Despite higher titer for RSV, IAV was more potent in increasing expression of IL-17a, IL-17c, IL-12b, IL-18, IL-23a, IL-1β, Ccl-2, and Ccl-7 than RSV. It is possible that cytokine expression was also increased in the poly(I:C) group, but emphysematous changes were not apparent. Although we did not compare cytokine in the lungs of this group of mice with the RSV and IAV groups, a study using CS-poly(I:C) mouse model showed that poly(I:C) was a potent stimulator of IL-18, IL-12/IL-23 p40, and type I and type II IFNs with significantly greater responses in mice exposed to CS compared with mice in FA. This study suggest that IL-18Rα and IFN-γ play critical role in the pathogenesis of the inflammation and remodeling that is induced by CS plus poly(I:C). IAV [43, 61] and RSV [62] have been shown to induce Th17-related cytokines. Future studies will elucidate the specific roles of the different cytokines and chemokines in IAV- or RSV-infected or poly(I:C) treated mice exposed to CS.

In mice, IL-17 is essential for the development of emphysema from long term CS exposure [49] by activating the IL-17 → Ccl-2 → MMP-9 → MMP-12 signaling axis [49]. Increases in IL-17-regulated genes and chemokines such as Ccl-2 and Ccl-7 are associated with COPD progression in humans [63–65]. This is consistent with our data showing that increased IL-17 expression was associated with increased expression of MMP-12 mRNA levels. Mouse models with MMP-12-deficient macrophages [51, 66] and pharmacological inhibition of MMP-12 [66] showed that MMP-12 activity plays an important role in the development of emphysema.

It has long been believed that, in cigarette smoke–induced COPD, the alveolar destruction and enlargement is a direct consequence of inflammation and the associated imbalance in the extracellular matrix protease and antiprotease response, which leads to degradation of the elastin [67]. However, despite differences in inflammatory responses, IAV and RSV infections led to similar extent of emphysema in the CS-exposed mice. Further, IAV and RSV infections significantly enhanced TUNEL positivity and upregulation of the MMP-12 mRNA to a similar extent. Also, despite increases in inflammation by poly (I:C) similar to RSV, extent of emphysema caused by poly (I:C) was milder. These findings suggests that inflammation does not necessarily correlate with lung pathology as was shown for CS-induced emphysema in C3H and C57Bl/6 mice [11]. It is also possible that regardless of extent of lung inflammation, the severity of alveolar destruction and airspace enlargement in mice remains mild [37], likely because of lung structure or length of chronic inflammation. While infection with RSV leads to differentiation of Th17 cells [62, 68] and is associated with skewing the immune system away from the Th1 response [69], poly (I:C) may cause more Th1 than Th17 differentiation [70, 71]. Furthermore, although poly (I:C) activates TLR3 [72], repeated doses of poly (I:C) induce inflammation and alveolar remodeling via pathways independent of TLR3 [28]. Future studies will investigate the role of innate versus Th17 immunity in the pathogenesis of IAV-, RSV-, and poly (I:C)-induced lung inflammation in CS-exposed mice.

Both weighted mean alveolar volume and Lm are useful parameters of peripheral lung structure and have utility in studying experimental emphysema [73, 74]. However, Lm reflects the dimensions of alveoli but is not a direct estimate of alveolar diameter or mean alveolar size. Although weighted mean alveolar volume, *L*m, and indexes of lung elasticity are correlated in excised non-diseased lungs of humans [75] and several animal species [76] Lm may not be the most accurate measure. Lm is a function of lung volume [76, 77] but may not be able to separate the effects of tissue destruction from those on tissue distension and from any changes in lung elasticity that results in changes in lung volume, as would be expected with emphysema. Thus, conclusions drawn from Lm alone should be interpreted with caution.

## Conclusion

Our studies demonstrate that IAV, RSV or poly (I:C) augment CS-induced airway and alveolar inflammation and remodeling in the murine lung. This study affirms that both RSV and IAV are key etiological factors driving cytokine, protease, and apoptotic responses that lead to airspace enlargements. However, there were significant differences in the type and extent of inflammation involved. The minimal emphysematous changes in the poly(I:C) group, compared to the CS-IAV and CS-RSV group, may be attributed to the differences in the study design and the shorter time elapsed between exposure to CS and analyses of the lung tissues. Future studies should elucidate the cause-effect relationships between inflammation and alveolar destruction in detail to dissect differences between these models to help translate these findings to patients with COPD exacerbations.

## Abbreviations

BAL, broncho-alveolar lavage; COPD, chronic obstructive pulmonary disease; CS, cigarette smoke; ES&H, Environmental Safety and Health department; FA, filtered air; GAPDH, glyceraldehyde-3-phosphate dehydrogenase; H&E, hematoxylin and eosin; IACUC, Institutional Animal Care and Use Committee; IAV, Influenza A Virus; Lm, mean linear intercept; mRNA, messenger ribonucleic acid; PCR, polymerase chain reaction; Poly(I:C), polyinosine-polycytidylic acid; RSV, respiratory syncytial virus; RT-PCR, reverse transcriptase polymerase chain reaction; SEM, standard error of the mean; TPM, total particulate matter; TUNEL, terminal deoxynucleotidyl transferase dUTP nick end labeling.
